# Determination of nutrients in seawater by segmented–flow analysis with higher analysis rate and reduced interference on ammonia

**DOI:** 10.1155/S1463924692000300

**Published:** 1992

**Authors:** M. Jodo, K. Kawamoto, M. Tochimoto, S. C. Coverly

**Affiliations:** 1 Tokyo Kyuei 6906-10 Tsurugamaru Shiba Kawaguchi Saitama-ken Japan; 2 Bran + Luebbe KK Shouei Bldg 22-8, 4-Chome Sendagaya, Shibuya-ku Tokyo 151 Japan; 3 Bran + Luebbe PO Box 1360 Norderstedt W-2000 Germany

## Abstract

A microbore continuous-flow analyser was used to determine ammonia, nitrite, nitrate and phosphate in seawater. Ninety samples per hour were measured by optimizing the hydraulic conditions and using a cadmium coil for nitrate reduction. The analysis range was extended and the detection limit was reduced by using two analytical ranges with automatic range-changing. Interference from magnesium on ammonia was investigated and reduced by using a different complexing agent. The results correlated well with a Japanese reference method.


Journal of Automatic Chemistry, Vol. 14, No. 5 (September–October 1992), pp. 163–167

### Determination of nutrients in seawater by segmented-flow analysis with higher analysis rate and reduced interference on ammonia

**M. Jodo**
*Tokyo Kyuei, 6906-10 Tsurugamaru Shiba Kawaguchi, Saitama-ken, Japan*

**K. Kawamoto, M. Tochimoto**
*Bran + Luebbe KK, Shouei Bldg, 22-8, 4-Chome, Sendagaya, Shibuya-ku, Tokyo 151, Japan*

**and S. C. Coverly***
*Bran + Luebbe, PO Box 1360, W-2000 Norderstedt, Germany*

*A microbore continuous-flow analyser was used to determine ammonia, nitrite, nitrate and phosphate in seawater. Ninety samples per hour were measured by optimizing the hydraulic conditions and using a cadmium coil for nitrate reduction. The analysis range was extended and the detection limit was reduced by using two analytical ranges with automatic range-changing. Interference from magnesium on ammonia was investigated and reduced by using a different complexing agent. The results correlated well with a Japanese reference method.*

### Introduction

Since the introduction of air-segmented continuous-flow analysis (CFA) in 1958 there have been continual improvements in system performance, mainly in analysis rate and the automation of activities subordinate to the analysis, such as automatic data handling, sample dilution and reagent change-over.

The analysis rate in segmented-flow systems is inversely dependent on dispersion. Reductions in dispersion have centred around reductions in the internal diameter of the reaction tubing, from 2·4 mm in the original Technicon AutoAnalyzer I to 2 mm in the AutoAnalyzer II and 1 mm in modern instruments. The latest systems have been designed with a proper understanding of the way in which dispersion, tubing diameter and flow rate are interrelated, following Snyder's definitive description [1]. As Snyder has also explained, a further reduction in dispersion in commercial systems is unlikely for practical reasons [2]. Segmented-flow systems run faster than flow-injection (FIA) systems operating at the highest possible sensitivity with slow reactions such as colorimetric ammonia and phosphate, due to the time needed to introduce a large injection volume into the manifold and the time required for the signal to return to near zero in a medium-dispersion FIA system.

A major source of dispersion in the determination of nitrate in seawater is the cadmium reduction stage, which commonly uses a column packed with cadmium granules. Patton [3] first described the use of a cadmium coil for analysing nitrate in fresh water. The coil allows the segmented stream to flow uninterrupted through the reduction stage. This more than doubled the analysis rate for seawater samples compared to methods using a packed column.

Previous experience using the AutoAnalyzer ammonia method [4] showed negative values in surface samples far from the coast. The method uses citrate and tartrate buffers to reduce magnesium interference similar to the method described by Hansen and Grasshof [5]. Experiments showed that this was not due to impurities in the standards and base-line reference solution, but, rather, to a suppression of the ammonia colour reaction due to magnesium. The complexing agents tartarte, citrate and EDTA were found to be effective against calcium but did not completely eliminate interference from magnesium. As described below, 1,2 cyclohexane diamine tetraacetic acid (CyDTA) proved to be a more effective complexing agent for magnesium.

To attain low detection limits in automated methods, it is necessary to use a high sample-to-reagent ratio, a flowcell with a long path-length and high signal amplification. This restricts the analytical range so that high samples have to be diluted. The range-expanding facility of the analyser is used to maintain a wide analytical range and achieve lower detection limits.

After optimizing the analysis conditions for nitrite, nitrate, ammonia and phosphate, the results from the automatic analyser were compared to those from a standard manual method [6]. The correlation ($R$) was greater than 0·99 for each parameter.

### Materials and methods

*Analyser*

A four-channel TRAACS 800 with random-access sampler and automatic dilution facility was used (Bran + Luebbe, Norderstedt, Germany).

*Reagents*

All chemicals were of analytical grade. Deionized water (<1 μs/cm) was passed through an activated carcoal column and a 0·4 μm filter. The numbers below in square brackets refer to the flow diagram (figure 1).

* Author to whom correspondence should be addressed.

0142–0453/92 $3.00 © 1992 Taylor & Francis Ltd.

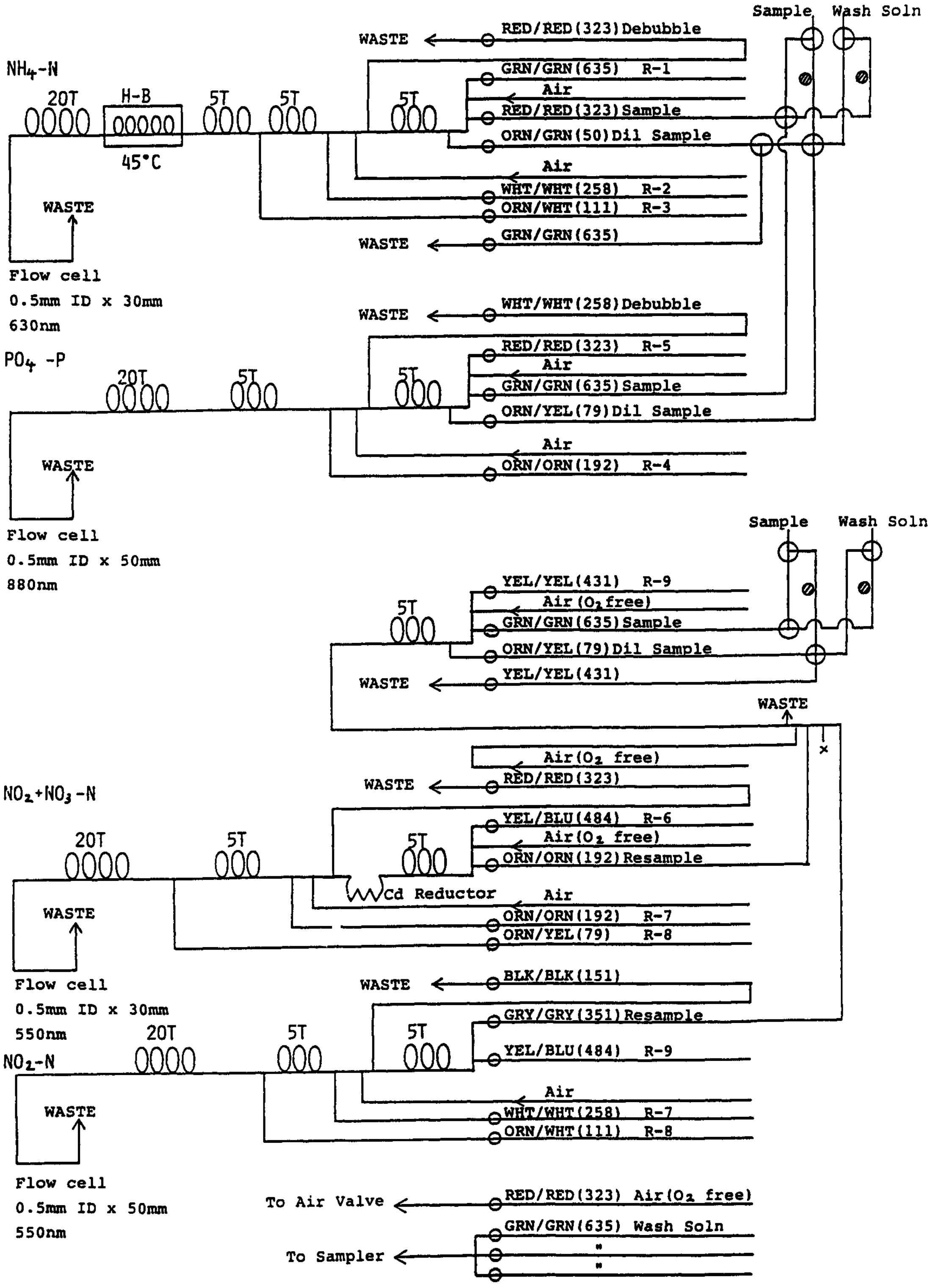

*Figure 1. Flow diagram of the system.*

*Ammonia*: Stock CyDTA solution

1 l contains:

5 g sodium hydroxide

5 g sodium carbonate

18 g 1,2 cyclohexane diamine tetraacetic acid (CyDTA).

Dissolve in the stated order, then adjust to pH 10.

Stock nitroprusside

1 l contains 0·6 g sodium nitroprusside (disodium nitrosylpentacyanoferrate-II dihydrate).

Working CyDTA [R1 on flow diagram]

Mix 240 ml stock CyDTA, 60 ml stock nitroprusside and 0·5 ml Triton X-100 (40% solution in ethanol or propan-2-ol).

Alkaline phenol reagent [R2]
1 l contains:
47·5 g phenol
95 ml 5M NaOH solution
Store in a plastic container in the refrigerator. Stable for one week.

Sodium hypochlorite [R3]
After determining the free chlorine content of commercially available hypochlorite solution by iodometric titration, prepare a working solution containing 1–1·5% chlorine.
Prepare fresh daily.

*Phosphate*:

Stock molybdate solution
1 l contains:
8 g sodium molybdrate dihydrate
0·17 g potassium antimony tartrate
50 ml sulphuric acid (conc.).

Working molybdate reagent [R4]
Add 0·8 g ascorbic acid and 2 ml 15% sodium dodecyl sulphate (purest grade) to 100 ml stock molybdate.
Prepare fresh daily.

Phosphate dilution [R5]
1 l contains:
30 g sodium chloride
10 ml sulphuric acid
5 ml sodium dodecyl sulphate (15% solution in water).

*Nitrate*:

Imidazole [R6]

1 l contains:

4 g imidazole

1 ml Triton X-100 (40% solution in propan-2-ol).

Adjust to pH 7·2.

Sulfanilamide [R7]
1 l contains:
10 g sulfanilamide
100 ml hydrochloric acid.

NEDD [R8]

1 l contains 1 g N-1-naphthylethylenediamine dihydrochloride.

Nitrate dilution water [R9]
1 l contains 3 ml Triton X-100 (40% in propan-2-ol).

*Standards*

Primary standards with a concentration of 10 mmol/l were prepared from ammonium sulphate, sodium nitrite, potassium nitrate and potassium dihydrogen orthophosphate. An intermediate standard containing ammonia (500 µmol/l, nitrite (100 µmol/l), nitrate (300 µmol/l) and phosphate (100 µmol/l) was prepared in 10% sodium chloride solution. This intermediate standard was diluted 20 to 200 times with water to produce the daily working standards.

*Analytical conditions*

The system was run at a sampling rate of 90/h and a sample : wash ratio of 4 : 1. Oxygen was removed from the air used for bubble segmentation on the nitrate channel by passing it through an oxygen absorber (activated iron, commercial hand-warmer available in Japan). An alternative would be to use nitrogen. This is necessary to avoid premature degradation of the reduction performance of the cadmium coil [3].

*Table 1. Results for seawater samples in µmol ammonia/l.*

| | Sample A | Sample B | Sample C |
|---|---|---|---|
| Tartrate method | −0·41 | −0·23 | −0·35 |
| CyDTA method | 0·11 | 0·35 | 0·21 |

*Table 2. Effect of different Mg levels on ammonia determination, with various NaOH concentrations in alkaline phenol reagent (phenol = 95 g/l). Results in µmol ammonia/l.*

| | NaOH 35 g/l | NaOH 40 g/l | NaOH 45 g/l |
|---|---|---|---|
| Tatrate method: | | | |
| Seawater, 55 mmol Mg/l | −0·36 | −0·05 | 0·30 |
| Standard, 30 mmol Mg/l | −0·37 | −0·24 | 0·01 |
| Standard, 60 mmol Mg/l | −0·57 | −0·45 | 0·10 |
| CyDTA method: | | | |
| Seawater, 55 mmol Mg/l | 0·29 | 0·23 | 0·24 |
| Standard, 30 mmol Mg/l | 0·13 | 0·02 | 0·03 |
| Standard, 60 mmol Mg/l | 0·23 | 0·14 | 0·12 |

*Table 3. Between-run reproducibility (N = 30).*

| Determinand | Concentration (µmol/l) | Mean (µmol/l) | SD (µmol/l) | CV % |
|---|---|---|---|---|
| Ammonia: | 0 | 0·0002 | 0·041 | — |
| | Sample | 0·87 | 0·044 | 5·0 |
| | 5 | 5·02 | 0·039 | 0·77 |
| | 10 | 9·98 | 0·062 | 0·62 |
| | 20 | 20·04 | 0·068 | 0·33 |
| Phosphate: | 0 | 0·0002 | 0·008 | — |
| | Sample | 0·22 | 0·007 | 3·2 |
| | 1·25 | 1·23 | 0·005 | 0·4 |
| | 2·5 | 2·47 | 0·006 | 0·24 |
| | 5 | 5·00 | 0·008 | 0·16 |
| Nitrate: | 0 | 0·008 | 0·033 | — |
| | Sample | 0·95 | 0·031 | 3·3 |
| | 5 | 5·02 | 0·027 | 0·53 |
| | 10 | 10·11 | 0·02 | 0·19 |
| | 20 | 20·26 | 0·04 | 0·19 |
| Nitrite: | 0 | −0·006 | 0·014 | — |
| | Sample | 0·3 | 0·013 | 5·7 |
| | 1·25 | 1·25 | 0·009 | 0·72 |
| | 2·5 | 2·51 | 0·007 | 0·27 |
| | 5 | 5·01 | 0·01 | 0·19 |

*Manifolds*

The flow diagram for all four channels is shown in figure 1. The cadmium reduction coil (Bran + Luebbe part No. 165-0301-01) was prepared and regenerated according to the manufacturer's instructions. The analysis concen-

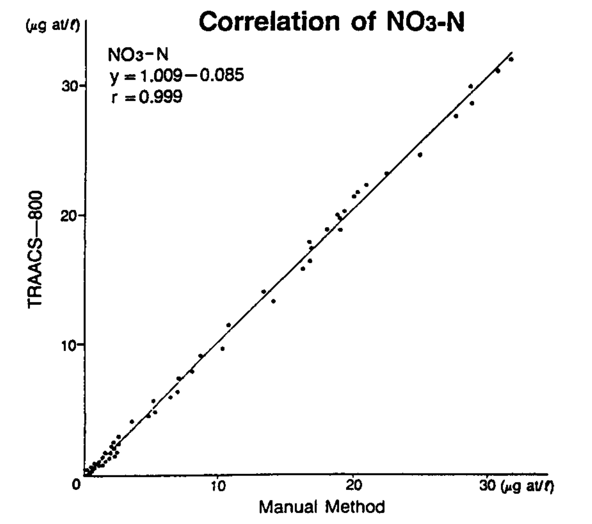

*Figure 2*

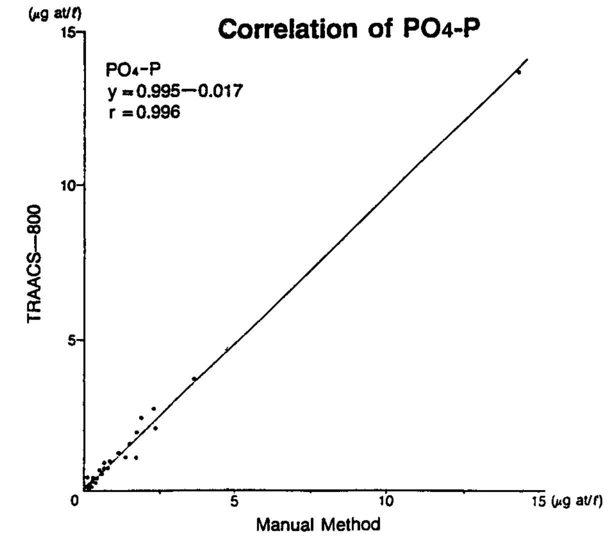

*Figure 4*

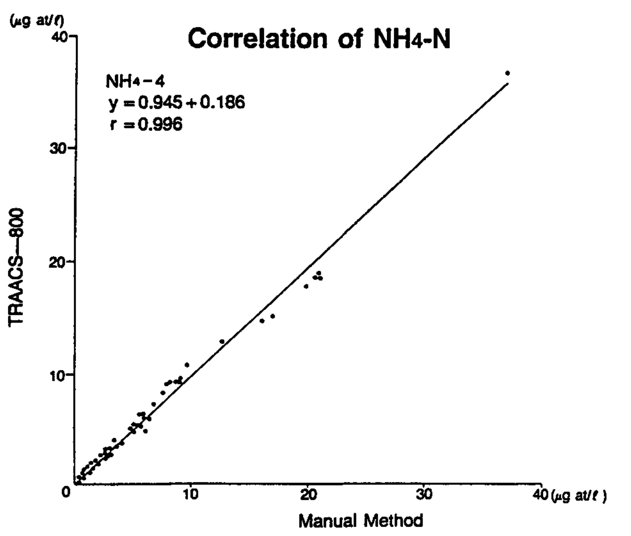

*Figure 3*

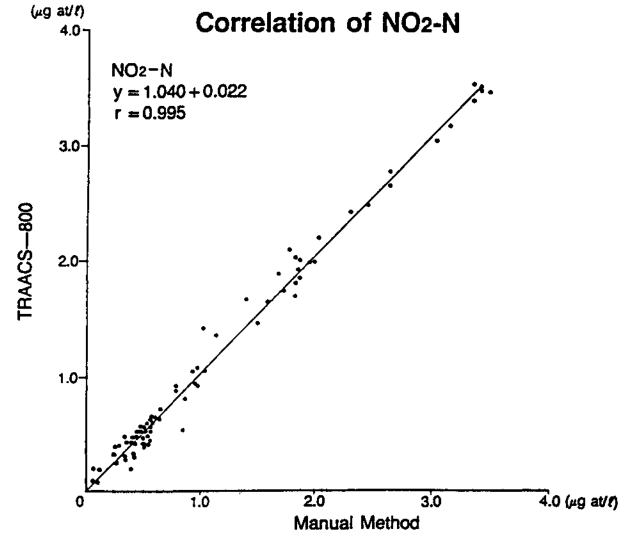

*Figure 5*

*Figures 2–5. Correlation of automated methods with reference methods.*

tration ranges in the low and high ranges were:

| | |
|---|---|
| Ammonia | 10 and 40 µmol/l |
| Nitrate | 10 and 40 µmol/l |
| Nitrite | 2 and 8 µmol/l |
| Phosphate | 4 and 16 µmol/l |

Each manifold has two sample pump tubes of different flow rates. In routine operation, samples were first analysed using the large pump tubes. If any samples fell off scale, the following sample was automatically re-sampled at the end of the analysis, to eliminate any effect of carry-over from the off-scale sample. At the end of the run, the off-scale samples, and high standards, were re-analysed with the small sample pump tubes, giving a higher dilution. This allowed samples falling into both ranges to be placed randomly into the sample tray without risk of inaccuracy. By splitting the analytical range like this a lower detection limit on the low range was attained.

### Results and discussion

The effects of reagent composition on magnesium interference in the ammonia determination were investigated. Table 1 shows the results for three open-sea samples with low ammonia content, which were measured using with the complexing reagent from the AutoAnalyzer method (citrate/tartrate) and with CyDTA. The magnesium content of the samples was 50–55 mmol/l. EDTA was also tested: the results are not shown, but lay between tartrate and CyDTA. The effect of magnesium on the ammonia determination could be due to interaction with sodium phenolate, which would cause a change in the resonant structure of the phenolate ion and a decrease in colour.

Table 2 shows the influence of magnesium on the original tartrate method and the CyDTA method at different NaOH concentrations. These results show that the

CyDTA method is less affected by variations in the NaOH concentration. This is important, because hypochlorite solutions, even from the same supplier, contain variable ratios of hypochlorite to hydroxide according to their age, and the quantity of the stock solution required to prepare the working reagent varies according to the free chlorine concentration.

The sensitivity of each method in the low-level analysis range, measured by the absorbance produced by the top standard, was as follows: ammonia 0·1 at 10 μmol/l, nitrate 0·23 at 10 μmol/l, nitrite 0·23 at 2 μmol/l, phosphate 0·19 at 4 μmol/l.

Reproducibility was measured by analysing five different concentrations of each analyte 10 times, in random order, on three separate days. There were thus 30 results at each concentration. The day-to-day relative standard deviation was less than 1% for all parameters at levels above 5 μmol/l. These results are summarized in table 3.

The detection limit, defined as three times the standard deviation of the zero samples in the above test, was 0·12 μmol/l for ammonia, 0·024 μmol/l for phosphate, 0·1 μmol/l for nitrate and 0.052 μmol/l for nitrite. Based on past experience, determining the detection limit by the EPA procedure for Method Detection Limit, which measures 10 or more consecutive low-level samples, would produce figures about half those above.

To validate the results from the automated methods, about 100 samples were collected from coastal areas around Japan and analysed according to the manual methods in the *Manual for Oceanographic Observation* [6], and on the automated system. The results showed good agreement, as shown in figures 2 to 5.

### Conclusions

By applying a flow-through cadmium reductor to a micro-bore segmented-flow analyser, reliable and reproducible results were achieved for nutrient analysis at a rate of 90 samples/h. CyDTA proved to be an effective complexing agent for magnesium, and reduced the negative effect on the ammonia determination. It also improved the robustness of the method to varying NaOH concentration. A disadvantage of CyDTA is its high cost.

By operating the analyser in the dual-range mode it was possible to reduce the detection limit and increase the analytical range by reducing the effect of carry-over on very low samples. This also improved the convenience of daily operation.

### References

1. Snyder, L. R., *Journal of Chromatography*, **125** (1976), 287–306.
2. Snyder, L. R., *Analytica Chimica Acta*, **114** (1980), 3–18.
3. Patton, C. J., Design, characterisation and applications of a miniaturized continuous flow analysis system. Doctoral thesis (Michigan State University, USA, 1982).
4. Technicon AutoAnalyzer II Method No. 154–71W.
5. Grasshoff, K., Ehrhardt, M. and Kremling, K., *Methods of Seawater Analysis* (Verlag Chemie, D-6940 Weinheim, 1983), 363–365.
6. *Manual for Oceanographic Observation* (Oceanographic Society of Japan, 1985).

